# A Robust Method for Inferring Network Structures

**DOI:** 10.1038/s41598-017-04725-2

**Published:** 2017-07-12

**Authors:** Yang Yang, Tingjin Luo, Zhoujun Li, Xiaoming Zhang, Philip S. Yu

**Affiliations:** 1 School of Computer Science and Engineering, Beihang University, Beijing, 100191 China; 2 Department of Computer Science University of Illinois at Chicago, Chicago, 60607 United States; 3 College of Science, National University of Defense Technology, Changsha, Hunan 410073 China; 4 Department of Computational Medicine and Bioinformatics, University of Michigan, Ann Arbor, MI 48109 USA

## Abstract

Inferring the network structure from limited observable data is significant in molecular biology, communication and many other areas. It is challenging, primarily because the observable data are sparse, finite and noisy. The development of machine learning and network structure study provides a great chance to solve the problem. In this paper, we propose an iterative smoothing algorithm with structure sparsity (ISSS) method. The elastic penalty in the model is introduced for the sparse solution, identifying group features and avoiding over-fitting, and the total variation (TV) penalty in the model can effectively utilize the structure information to identify the neighborhood of the vertices. Due to the non-smoothness of the elastic and structural TV penalties, an efficient algorithm with the Nesterov’s smoothing optimization technique is proposed to solve the non-smooth problem. The experimental results on both synthetic and real-world networks show that the proposed model is robust against insufficient data and high noise. In addition, we investigate many factors that play important roles in identifying the performance of ISSS.

## Introduction

Almost everything in our daily life can be modeled as complex networks, such as the social network^1, 2^, transportation network^3^, protein-to-protein network^4^ and knowledge graph^5^. The interactions in these networks play an important role in identifying the networks’ structure, functionality and dynamics. Hence, many researchers utilize the detailed interaction information to do a lot of interesting research works, such as the centrality measures^6, 7^, community detection^8^, robustness analysis^9–11^, controllability^12^ and network game^13–15^. However, the link information of networks is often invisible in many cases. Hence, it’s very important to propose a robust method in inferring the network structure from very few observed values (measurements) with noise, which are indirectly generated from the network.

Many researchers have made great efforts in solving the network inference problem. For instance, Han and Di *et al*.^16^ proposed the state-of-art method i.e., Lasso (Least Absolute Shrinkage and Selection Operator)^17^, which provides an estimation of a network with limited connectivity and low model prediction error. Hempe^18^ proposed an inference algorithm based on inner composition alignment to identify the network structure^19, 20^ on the time series data^21^. Timme^22^ and Domenico Napoletani *et al*.^23^ inferred the complete connectivity of a network from its stable response dynamics. Daniel Marbach *et al*.^24^ summarized many robust gene network inference methods and compared their performances. Soheil Feizi *et al*.^25^ proposed network deconvolution as a general method to distinguish direct dependencies in gene expression regulatory networks.

Though many researchers have made great efforts in solving the network inference problem, it is still very challenging mainly on the following reasons. Firstly, the interactions in the network are very sparse. For most of the vertices of the network, they have few interactions with their neighbors, which are only a small portion of vertices compared with the network size, i.e. the total number of vertices. It leads to the sparseness of interactions for each vertex, which makes it hard to infer the local structure. Secondly, the inference of the inaccessible original network is mainly based on the observations, i.e. measurements generated from the network. However, almost all of measurements and outputs have noises. The noisy data present great challenges in the error control, and it creates great difficulties to identify the real network. Thirdly, the observations are always insufficient i.e., have missing terms. In many real world applications, it’s hard or costly to get all the measurements of the network from the beginning to the end. For many real world cases, we have only 5–20% observations^16^ on the whole network, which makes it an impossible task to infer the detailed network structure.

In this paper, we propose an iterative smoothing algorithm with structure sparsity (ISSS) method to infer the network structure. The elastic penalty is the combination of *L*_1_ and *L*_2_ penalties. The special *L*_1_ penalty can successfully model the sparseness of network information. The *L*_2_ penalty can prevent over-fitting of the model. The *TV* penalty^26^ of the method can treat the neighbors of each vertex as a continuous signal, which makes the model tend to infer the local information of the network, i.e. community structure. The three structural penalties lead to the non-smoothness of the loss function, which makes it hard to solve the optimization problem. By introducing the Nesterov’s smoothing technique^27^, the optimization problem can be successfully solved. Moreover, the existence of three penalties and the smoothing technique makes the inference method more robust, even when the network has an insufficient observation with noise. We apply the model on synthetic networks to infer the dynamics of these networks, such as Erdős Rényi (ER)^28^, Barabási Albert (BA)^29^ and Watts Strogatz (WS)^30^ networks. Furthermore, we also apply the model on many real world networks, such as the Karate^31^ and Netscience^32^ networks. The experimental results show the effectiveness of the ISSS model.

## Problem Definition

Let’s take the ultimatum game (UG) on networks^33^ as an example. The vertices in the network represent players. If two people play the ultimatum game, there is an edge between them. Everyone in the game will play two times with his neighbors using strategy (*p*, *q*), both as a proposer and a responder. *p*_*ij*_ ∈ [0, 1] is the amount proposed by player *i* to offer his neighbor *j*, while *q*_*ij*_ is the minimum amount that *i* responds to his neighbor *j*. For instance, player *i* has a strategy [0.8, 0.65], and one of his neighbors *j* has a strategy [0.6, 0.5]. Since *i* proposes to offer *p*_*ij*_ = 0.8 to *j*, which is larger than the minimum acceptance amount of *j*, i.e., *q*_*ji*_ = 0.5, then player *i* will get a 1−0.8 = 0.2 payoff. At the same time, *j* offers *i* with *p*_*ji*_ = 0.6, which is lower than the minimum amount of *i* accepts, i.e., *q*_*ij*_ = 0.65, then player *i* rejects *j*’s offer, and both of them get nothing. The game lasts for *M* rounds. For round *μ* ∈ [1, *M*] with time stamp *t*_*μ*_, the corresponding strategy of player *i* is denoted as (*p*_*j*_(*t*_*μ*_), *q*_*j*_(*t*_*μ*_)). The payoff of player *i* with player *j* at round *μ* can be summarized as follows:

1 $${\varphi }_{ij}({t}_{\mu })=\{\begin{array}{ccc}{p}_{j}({t}_{\mu })+1-{p}_{i}({t}_{\mu }) & {p}_{i}({t}_{\mu })\ge {q}_{j}({t}_{\mu })\,and & {p}_{j}({t}_{\mu })\ge {q}_{i}({t}_{\mu })\\ 1-{p}_{i}({t}_{\mu }) & {p}_{i}({t}_{\mu })\ge {q}_{j}({t}_{\mu })\,and & {p}_{j}({t}_{\mu }) < {q}_{i}({t}_{\mu })\\ {p}_{j}({t}_{\mu }) & {p}_{i}({t}_{\mu }) < {q}_{j}({t}_{\mu })\,and & {p}_{j}({t}_{\mu })\ge {q}_{i}({t}_{\mu })\\ 0 & {p}_{i}({t}_{\mu }) < {q}_{j}({t}_{\mu })\,and & {p}_{j}({t}_{\mu }) < {q}_{i}({t}_{\mu })\end{array}$$

**Figure 1:**
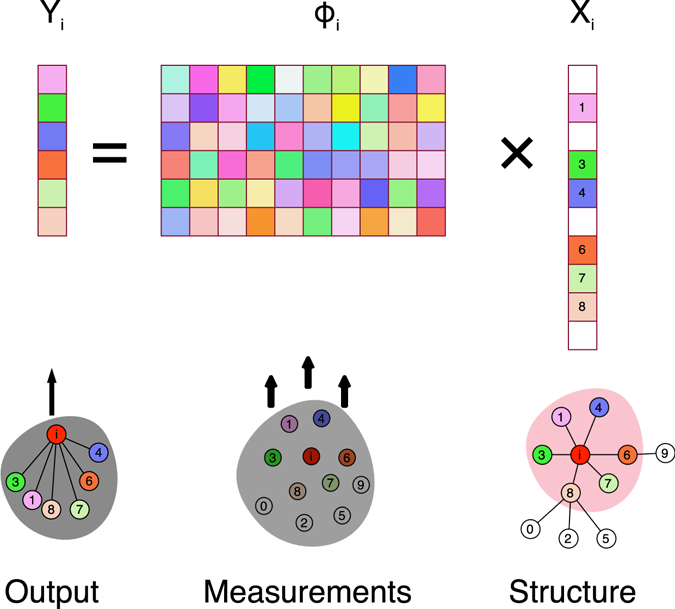
The network structure inference problem. The colorful squares in *Y*_*i*_ and Φ_*i*_ represent different float values. The colorful squares in *X*_*i*_ represent the connection between vertex *i* and other vertices. If the square of vertex *j* is blank, it means that there is no connection between vertex *i* and *j*. Hence, the color of squares in *X*_*i*_ is the same with the corresponding color of nodes in the graph. In many real world applications, the training data is insufficient, which indicates that some rows of *Y*_*i*_ and Φ_*i*_ are missing, such as the *j* ∈ *E*_*missing*_ row in *Y*_*i*_ and Φ_*i*_.

Hence, the overall payoff of player *i* with all its neighbors is defined as ${y}_{i}({t}_{\mu })={\sum }_{j\in {E}_{i}}{\varphi }_{ij}({t}_{\mu })$, where *E*_*i*_ is the neighbors of player *i*. The strategy (*p*_*i*_(*t*_*μ*+1_), *q*_*i*_(*t*_*μ*+1_)) changes from time to time with a random number *δ* ∈ [*−ε*, *ε*]. *ε* is set to 0.05 and the strategy *p* and *q* are set to range [0, 1]. The reconstruction problem is formulated as *Y*_*i*_ = Φ_*i*_ × *X*_*i*_ as shown in Fig. 1, where ${Y}_{i}\in {{\mathbb{R}}}^{M\times 1}$ is given by the output of the network system for vertex *i*. ${{\rm{\Phi }}}_{i}\in {{\mathbb{R}}}^{M\times N}$ is the observation, i.e. measurements which is constructed by Equation (1). ${X}_{i}\in {{\mathbb{R}}}^{N\times 1}$ denotes the neighboring vector of vertex *i*, which is the network structure we want to infer_._ *t*_*μ*_ ∈ [1, *M*] is the accessible time instance in time series. Our task is to infer all the neighbors of vertex *i*, i.e., *X*_*i*_, given noisy, sparse and incomplete *Y*_*i*_ and Φ_*i*_ in Equation (2).

2 $$[\begin{array}{c}{y}_{i}({t}_{1})\\ {y}_{i}({t}_{2})\\ \mathrm{.}\\ \mathrm{.}\\ \mathrm{.}\\ {y}_{i}({t}_{M})\end{array}]=[\begin{array}{cccc}{\varphi }_{i1}({t}_{1}) & {\varphi }_{i2}({t}_{1}) & \mathrm{...} & {\varphi }_{iN}({t}_{1})\\ {\varphi }_{i1}({t}_{2}) & {\varphi }_{i2}({t}_{2}) & \mathrm{...} & {\varphi }_{iN}({t}_{2})\\ \mathrm{.} & \mathrm{.} & \mathrm{.} & \mathrm{.}\\ \mathrm{.} & \mathrm{.} & \mathrm{.} & \mathrm{.}\\ \mathrm{.} & \mathrm{.} & \mathrm{.} & \mathrm{.}\\ {\varphi }_{i1}({t}_{M}) & {\varphi }_{i2}({t}_{M}) & \mathrm{...} & {\varphi }_{iN}({t}_{M})\end{array}][\begin{array}{c}{x}_{i1}\\ {x}_{i2}\\ \mathrm{.}\\ \mathrm{.}\\ \mathrm{.}\\ {x}_{iN}\end{array}]$$

Here the UG on networks is just an example. There are also many other applications, such as the evolutionary games, transportation, communication processes, sociology and biology in papers^16, 34, 35^. For instance, the construction of biological interaction networks with the goal of uncovering causal relationships between genotype and phenotype constitutes a major research topic in systems biology^35^. With the increased availability of DNA microarray time-series data, it is possible to infer gene regulatory networks (GRNs) from gene expression data to understand dynamic GRNs^36^. The information-geometric network inference to use a real radio emulation testbed to infer the end-to-end rate distributions of stochastic network flows from link rate measurements^37^. G Zahoranszky-Kohalmi^38^ employs network inference method in investigating the drug-target and target-target interactions in order to design new drugs.

In these real world applications, the observations from the network, i.e., Φ_*i*_ and *Y*_*i*_ have noises, and Φ_*i*_ is generally sparse and incomplete, which are key factors in identifying the performance of the network inference method. In fact, the essence of the inferring network structure (network reconstruction) problem can be formulated by Equation (2). The performance of the model is determined by the network structure and the expressing ability of model itself. Thus if the networks are built using ER network model with the same parameters, then the performance of any methods should be very similar. In the next section, we will discuss the advantages of ISSS and the key factors in identifying the performance of the ISSS and Lasso models.

## Results

In this section, we apply our model on many different types of synthetic and real world networks. Then we compare the experimental results of our model with the state-of-art method Lasso^16^ on ER, BA and WS networks. The experimental results show the effectiveness of our model, and it outperforms the baseline method significantly. In addition, we also investigate the key factors in identifying the performance of the model, such as the variance of the elements in measurement Φ and the properties of many real world networks. Finally, we compare the clustering coefficients of the network derived from the Lasso and ISSS model, respectively. And we also analyze the effectiveness of three penalties in the model.

### Experiments on three typical networks

We take the network inference as a binary classification task, i.e. the edge exists or not. Hence, Area under the Receiver Operating Characteristic (AUROC)^39^ is employed to evaluate the performance of the models. The Area under the Receiver Operating Characteristic is a common summary statistic for the goodness of a predictor in a binary classification task. It is equal to the probability that a predictor will rank a randomly chosen positive instance higher than a randomly chosen negative one. According to the experimental results in Table 1, the ISSS method outperforms Lasso on all the ER, BA and WS networks with different training data size, which is defined as *Data* = *M/N*, where *M* is the number of accessible time instances in the time series and the *N* is the number of vertices in the network. In the experiment, the ER, BA and WS networks all have *N* *=* 100 vertices, *M* = 100 time instances and the edges of networks are constructed randomly according to the parameters. All the experimental results are averaged over at least 10 independent trials. The experiment setup in this paper is the same with the sate-of-art Lasso. With more than 40% of the training data, i.e. *Data* = 0.4, both ISSS and Lasso can successfully identify the network structure. As shown in Table 1, when the training data size is very small, the performance of ISSS is much better than Lasso. Take *Data* = 0.05 as an example, the AUCROC of ISSS is at least 0.1 larger than that of Lasso. It indicates that the ISSS improves the performance significantly with very few training data.

**Table 1:** Comparison between ISSS and Lasso.

Type	Method	Data				
		0.05	0.1	0.15	0.2	0.4
ER	Lasso	0.51	0.55	0.59	0.64	0.95
	ISSS	**0**.**63**	**0**.**71**	**0**.**76**	**0**.**80**	0.95
BA	Lasso	0.53	0.63	0.81	0.92	0.99
	ISSS	**0**.**73**	**0**.**79**	**0**.**86**	**0**.**92**	1.00
WS	Lasso	0.53	0.60	0.71	0.87	1.00
	ISSS	**0**.**71**	**0**.**79**	**0**.**86**	**0**.**93**	1.00

We use AUROC^39^ (Area under the Receiver Operating Characteristic) index to depict the performance of the inference task. Area is the area under the curve. It is a common summary statistic for the goodness of a predictor in a binary classification task. It is equal to the probability that a predictor will rank a randomly chosen positive instance higher than a randomly chosen negative one.

**Figure 2:**
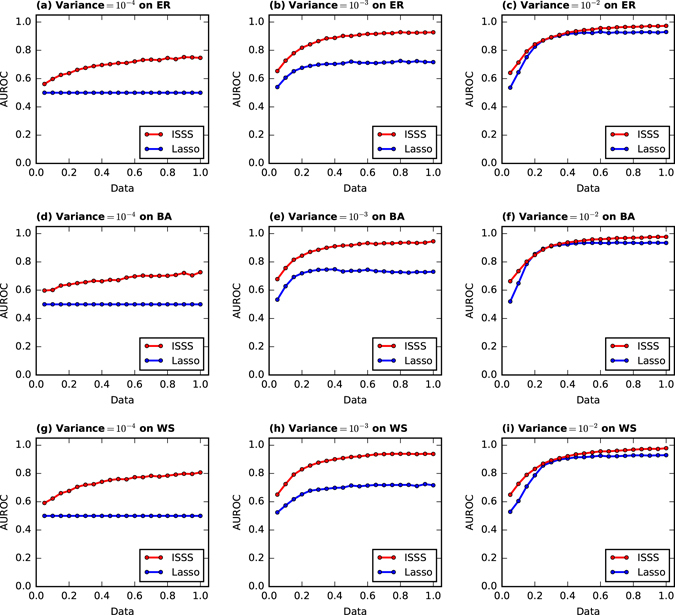
The performance of ISSS and Lasso on the data with *variance* = 10^−2^, 10^−3^ *and* 10^−4^. With very small *variance* = 10^−4^, the performance of Lasso is equal to random guess, i.e., the blue line in Fig. 2(a,d,g). With very large variance, the inference or reconstruction task is much easier. To show the whole curves, the upper bound of the y axis is set to 1.2 for all the figures in this paper.

It is worth mentioning that variance of measurement Φ’s elements in Equation (2) play an important role in inferring the network structure. The Φ’s elements Φ_*ij*_ ∈ [0, 1] on ER, BA and WS in Table 1 has default *variance* = 10^−1^. Here we will analyze the situation that Φ has many other variances, s.t. *variance* = 10^−2^, 10^−3^ and 10^−4^ in Fig. 2. We plot the performance of ISSS and Lasso with different variances of Φ’s elements on ER, BA and WS networks.

On the networks with *variance* = 10^−4^ in Fig. 2(a,d,g), i.e. the network structure is hard to reconstruct, Lasso fails to infer the network structure. The performance of Lasso is the same with the random guess. Compared with the Lasso, ISSS method greatly outperforms the lasso significantly. As the size of training data goes up, the performance of ISSS gets better. However, the AUROC of ISSS is still lower than 0.8 with all the training data, i.e., *Data* = 1. On the networks with *variance* = 10^−3^, as shown in Fig. 2(b,e,h), there is a big gap between two curves, which indicates that ISSS greatly outperforms the Lasso. With *variance* = 10^−2^ as shown in Fig. 2(c,f,i), the performance of ISSS is greatly improved especially when we have only 5% training data. As the size of training data rises, both the performances of ISSS and Lasso are improved. An interesting phenomenon is that ISSS and Lasso perform similarly among 25–40% training data with *variance* = 10^−2^. The cause of the phenomena is hard to interpret from theory and experiment aspects. In addition, low variance leads to faster convergence of the model. For instance, to infer a small network, the training time with *variance* = 10^−3^ is 10% shorter than that with *variance* = 10^−1^. That’s the reason that many systems prefer low variance measurements and outputs. In summary, the ISSS model performs well with low variance measurements and few training data.

**Figure 3:**
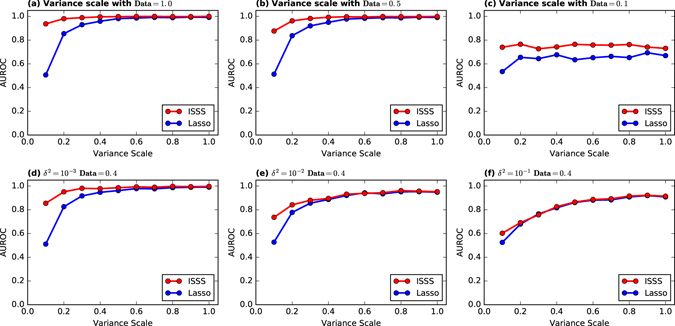
Insufficient data vs variance scale. In most cases, the AUROC of ISSS and Lasso rises, as the variance increases. With small variance, ISSS performs much better than the Lasso. With *Data* = 0.1, the performance cannot be improved with any variance. It indicates that insufficient data plays an important role in the inference task. Extremely insufficient data leads to the failure of all the methods. The experimental results with other *Data* ∈ [0.2, 1] values are similar. In this section, we take *Data* = 0.4 as a representative. As shown in this figure, with small noise, ISSS outperforms Lasso. With great noise, the performance of ISSS and Lasso reach the upper bound of the model. Hence the performance of the two methods are the same.

To further investigate the relation between variance and training data size, as shown in Fig. 3(a,b,c), we fix the training data size, i.e., *Data* = 0.1, 0.5 and 1, then find out the performance of ISSS and Lasso as variance scale goes up from (0, 1] with step 0.1. The experimental results on ER, BA and WS network are similar. Hence, we take ER network as an example in the figure. When *Data* = 1 and 0.5, ISSS can successfully infer the network structure with very small variance scale. With *Data* = 0.1, the AUROC keeps its value as the variance goes up. The performance of Lasso is just a little better than the random guess. However, the AUROC value of ISSS is larger than 0.70. It indicates that training data size is an important factor in identifying the upper bound of the performance.

To test the robustness of the ISSS against the noise, we add some Gaussian noises ${\mathscr{N}}\sim (\mu ,{\delta }^{2})$ to the output *Y* and measurement Φ. Then we apply the ISSS and Lasso model on the noisy data. The results are shown in Fig. 3(d,e,f). For low noise with *δ* ^2^ = 10^−3^, the ISSS outperforms the Lasso, especially when the elements of Φ have very small variance. For very high noise that follows the normal distribution with *δ* ^2^ = 10^−1^, the performance of ISSS and Lasso are similar. For the noise with *δ* ^2^ = 10^−2^, the performance of ISSS is better than Lasso for all variance scales.

### Experiments on real world networks

**Figure 4:**
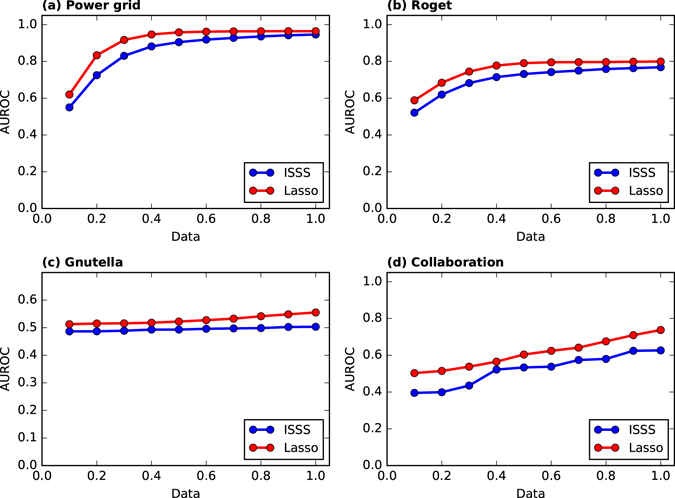
The performance of ISSS and Lasso on power grid, roget, Gnutella peer-to-peer file sharing network and collaboration network in computational geometry. The performance of ISSS and Lasso ON Gnutella network are not good enough. The reason is that we need more training data to infer the network, i.e., *Data* = *M/N* = 2, where *M* is the time instances and *N* is the number of vertices in the network.

We apply the ISSS and Lasso on many real world networks to evaluate the performance. Table 2 denotes the minimum data for achieving at least 0.95 AUROC in combination with several real world networks. Lasso cannot achieve 0.95 AUROC on all networks with default *variance* = 10^−1^. ISSS greatly outperforms the Lasso. On karate and Les Miserables networks, ISSS achieves 0.95 AUROC with only *Data* = 0.2 and 0.4, respectively. By analyzing many properties of the network, we find that it is much easier to infer the network structure with higher centralization index with very few training data. The network with both higher heterogeneity and density can also be easily inferred. To evaluate the performance of ISSS and Lasso on large networks, we do some experiments on the power grid (4941 vertices, 6594 edges), roget (1022 vertices, 5075 edges), Gnutella peer-to-peer file sharing network (6301 vertices, 20777 edges) and collaboration network in computational geometry (7343 vertices, 11898 edges). As shown in Fig. 4, ISSS outperforms the Lasso on all the networks. The Gnutella peer-to-peer network is hard to infer. Both ISSS and Lasso require more training data to infer the Gnutella peer-to-peer network structure.

**Table 2:** Minimum data for achieving at least 0.95 AUROC in combination with several real networks.

Network	Vertices	Eedgs	CC	Average path	Centralization	Heterogeneity	Density	Lasso	ISSS
Karate	34	78	0.571	2.408	0.4	0.833	0.139	N/A	0.2
Dolphins	62	159	0.259	3.357	0.116	0.572	0.084	N/A	0.5
Netscience	1589	2743	0.638	5.823	0.019	1.005	0.002	N/A	0.8
Adjnoun	113	435	0.173	2.536	0.38	0.903	0.068	N/A	0.6
Football	115	613	0.403	2.508	0.012	0.083	0.094	N/A	1
Les Miserables	77	254	0.573	2.641	0.397	0.91	0.087	N/A	0.4

CC stands for clustering coefficient.

**Figure 5:**
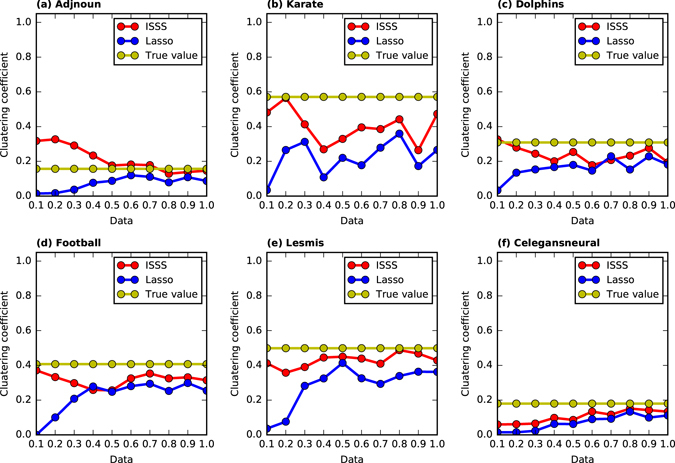
The clustering coefficient of the reconstructed network by ISSS and Lasso. The yellow line represents the real clustering coefficient of the network. The red line represents the clustering coefficient of the reconstructed network by ISSS.

**Figure 6:**
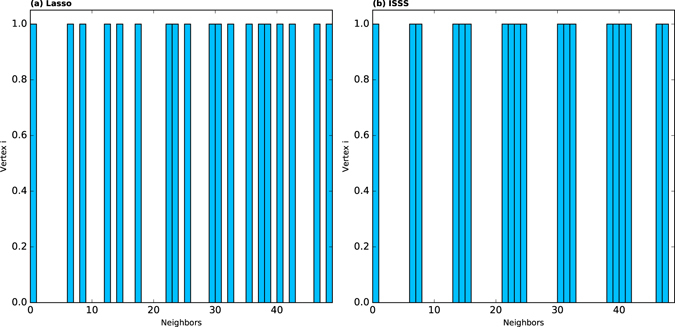
The reconstruction of vertex *i*’s neighbors. Suppose there are 50 vertices in all. If vertex *i* is connected with vertex *j*, the corresponding item in histogram is 1, otherwise it is 0. Lasso is good at predicting the neighbors in a discrete style, while the ISSS is inclined to predict continuous neighbors. The continuous neighbors are probably the way to construct the community structure of the network.

We compare the clustering coefficient of the network inferred by ISSS and Lasso with different training data sizes in this part. In Fig. 5, the horizontal axis represents the training data size, and the vertical axis is the clustering coefficient of the network inferred by ISSS and Lasso. The red solid line lies above the blue line all the time. It indicates that the ISSS model is inclined to infer the networks with stronger community structures than that of Lasso. As the training data size increases in Fig. 5, the clustering coefficient of the network inferred by ISSS approaches the ground truth, i.e., the yellow line. These results show that the TV penalty in ISSS model works well. Here is an example to demonstrate why the TV penalty works. For instance, a network has 50 vertices. As shown in Fig. 6, the horizontal axis represents all the 50 vertices in the network. If vertex *j* is a neighbor of vertex *i*, it is 1, otherwise it is 0 in the figure. Lasso tends to predict the neighbors of vertex *i* discretely as shown in Fig. 6(a), while the ISSS is opt to infer the network structure in a continuous way, as shown in Fig. 6(b). The reason for choosing TV penalty is that almost all of the vertices in many networks have similar natural number. Take the karate network as an example. Almost all the vertices’ labels that are less than 20 are in the same community. For the dolphin network, the vertices’ labels under 30 are connected with each other. For the networks don’t have natural number labels or the vertices don’t have continuous labels as shown in Fig. 6(a). We can remove the TV penalty from the model. The experimental results are shown in Table 3. The experiment is done on a BA network with continuous natural number labels. Without TV penalty, the ISSS model also works well. However, the ISSS with all the three penalties is still the best.

**Table 3:** The performance of models with different penalties on a BA network.

Data	0.05	0.10	0.15	0.20	0.4
ISSS	0.728	0.771	0.864	0.931	0.991
ISSS/TV	0.720	0.751	0.857	0.926	0.986
ISSS/L1	0.723	0.742	0.807	0.921	0.995
ISSS/L2	0.703	0.772	0.865	0.931	0.992
Lasso	0.542	0.587	0.677	0.757	0.941

ISSS/TV is the ISSS model without TV penalty. ISSS/L1 is the ISSS model without L1 penalty. ISSS/L2 is the ISSS model without L2 penalty.

As we mentioned above, TV penalty is a factor in inferring the network structure. Hence, how to choose the value of parameter *γ* is important as shown in Equation (4). In this part, we investigate the relationship between the most common used hyper parameter coefficients *γ* and the training data size, i.e. *Data*. According to the results in Table 4, *γ* = 10^−3^ and 10^−4^ have the best performance on almost all training data. Given *Data* = 0.1, *γ* = 10^−6^ is the best choice. The hyper parameter *β*, *λ* of *L*_1_, *L*_2_ are empirically set as 0.001 and 0.0001, respectively.

**Table 4:** Sensitivity analysis of TV penalty coefficient *γ* in Equation (4).

TV coefficient	Data				
	0.1	0.3	0.5	0.8	1
1.00E-06	0.8000	0.9700	0.9988	1.0000	1.0000
1.00E-05	0.8037	0.9750	0.9990	1.0000	1.0000
1.00E-04	0.7790	0.9815	0.9994	1.0000	1.0000
1.00E-03	0.7078	0.9830	0.9993	1.0000	1.0000
1.00E-02	0.7785	0.9538	0.9909	0.9996	0.9999
1.00E-01	0.7090	0.7832	0.8029	0.8363	0.8619
1.00E-00	0.4825	0.6137	0.6225	0.6406	0.6385

## Discussion

In this paper, we propose the robust ISSS model in solving the network reconstruction problem. Compared with the state-of-art Lasso, the ISSS model is more robust, which can successfully infer the network structure with insufficient training data against noise. The sparse regularization term *L*_1_, *L*_2_ and the structural *TV* penalties make the ISSS model reconstruct the network with strong community structure. The experiments on the WS, ER and BA networks show that the ISSS outperforms Lasso even when the network system has very few observations with great noises. We also do some experiments to analyze the factors in identifying the performance of the ISSS, i.e. the variance of Φ, training data size and the clustering coefficient of the network.

In the future, on the one hand, we will continue to find out all the factors in identifying the upper and lower bound of ISSS’s performance, and prove that from both theory and experiment perspectives. On the other hand, the community structure of a network defines the characteristic of a network, which identifies its functionality and should be preserved during the inferring process. Though ISSS model can identify the network with community structure, there could be room for improvement. For instance, the group Lasso and the tree group Lasso are the promising directions in network reconstruction task. Furthermore, we will optimize ISSS and apply the network inference method to solve the drug prediction and breast cancer prediction problems.

At present, it is still very challenging to infer many large-scale networks. Hence, it is a future trend with promising application field. And the evaluation criteria on directed and undirected networks are the same. As the development of network inference and compressed sensing research, more reasonable evaluation methods will be proposed to evaluate the experimental results on undirected networks.

## Methods

The main goal of complex network reconstruction is to recover the connection **X**_*i*_ by the payoff information *y*_*i*_ and virtual-payoff matrix Φ_*i*_ of vertex *i* from the time series of strategies. Note that the number of the neighbors of vertex *i* is often much less than the total number of vertices in the entire complex network, i.e., **X**_*i*_ is sparse. Han *et al*.^40^ adopted to use Lasso model to identify the neighbors of vertex *i*. By using Lasso model, the problem of network reconstruction is formulated as

3 $$\mathop{\min }\limits_{{{\bf{X}}}_{i}}{\Vert {Y}_{i}-{\Phi }_{i}{{\bf{X}}}_{i}\Vert }_{2}^{2}+\lambda {\Vert {{\bf{X}}}_{i}\Vert }_{1},$$

where *λ* is a non-negative regularization parameter. All nonzero elements of the reconstructed **X**_*i*_ correspond to the neighbors of *i* vertex. However, the Lasso-based model (3) only considers the sparse information.

In practice, to estimate a true signal in noise, the most frequently used methods are based on the total variation (TV) norm and the ${\ell }_{1}$ norm. TV penalty forces sparsity on the spatial derivative of the weight map. TV norms are essential ${\ell }_{1}$ norms of derivatives, hence ${\ell }_{1}$ estimation procedures are more appropriate for the subject of TV estimation. The space of functions of bounded total variation plays an important role when accurate estimation of discontinuities in solutions is required^26^. By contrast, total variation is remarkably effective at simultaneously preserving edges whilst smoothing away noise in flat regions for image denoising. Therefore, we consider to use ridge regression model with the TV regularization to preserve the structural information of networks and enhance the robustness performance of network reconstruction. After imposed the TV regularization, the objective of our method is formulated as

4 $$\mathop{{\rm{\min }}}\limits_{{{\bf{X}}}_{i}}F({{\bf{X}}}_{i})\mathop{=}\limits^{\Delta }{\Vert {Y}_{i}-{\Phi }_{i}{{\bf{X}}}_{i}\Vert }_{2}^{2}+\frac{\beta }{2}{\Vert {{\bf{X}}}_{i}\Vert }_{2}^{2}+\lambda {\Vert {{\bf{X}}}_{i}\Vert }_{1}+\gamma TV({{\bf{X}}}_{i}),$$

where *TV*(·) represents the TV regularization, which is defined as $TV({{\bf{X}}}_{i})={\sum }_{j=1}^{N-1}|{{\bf{X}}}_{i}(j+1)-{{\bf{X}}}_{i}(j)|$. It is worth noting that the Lasso-based model^40^ is a special case of our model. When *β* = 0 and *γ* = 0, our model will reduce to the Lasso-based model^40^ for complex network reconstruction.

Compared to the model in ref. 40, our model in (4) is able to utilize the structural information to help recover the connection of network. However, the TV regularization is a complex and non-smoothed sparsity-inducing penalties, and its proximal operator is unknown and difficultly computed. The traditional convex optimization methods, such as iterative soft-thresholding algorithm (ISTA^41^, fast or accelerated ISTA (FISTA)^42^ and first-Order primal-dual(Primal-Dual) algorithm^43^ and second-order methods^44–46^, require only one non-smoothed term alone or access to the unknown proximal operator of the non-smooth part of the objective function. In general, the proximal operator of the sum of two non-smooth functions, i.e. ${\ell }_{1}$ and *TV*, is unknown and computing expensive. Thus, we can not use them solve this problem (4) directly. Motivated by Nesterov’s smoothing technique^27^, we first smooth the TV penalty with the unknown proximal operator, while keeping the exact ${\ell }_{1}$ constraint and then use accelerated proximal gradient descent to minimize the whole function as shown in Algorithm 1.

Time-complexity of iterative convex optimization is kind of tricky to analyze, as it depends on a convergence criterion. Here we give a brief estimation of the time complexity. Suppose we have *N* players, and *M* time instances, as shown in Equation 2. The optimization procedure of the ISSS is shown in Algorithm 1. The first 3 setps are the most time-consuming, which costs *O*(*T*(*M* * *N* + *N* ^2^)), in which *T* is the number of iterations. For all *N* vertices, the time complexity of the ISSS is *O*(*T*(*M* * *N* ^2^ + *N* ^3^)). Lasso is implemented using LARS algorithm. (1) For *N* < *M*, *N* ^3^ < *N* ^2^ * *M* and the computational complexity of Lasso is *O*(*N* ^2^ * *M*). (2) For *N* ≥ *M*, *N* ^3^ ≥ *N* ^2^ *M and the complexity of Lasso is *O*(*N* ^3^). With limited *T* iterations, the time complexity of ISSS is slightly higher than that of Lasso.

### Nesterov’s smoothing technique for TV penalty

Denote $TV({{\bf{X}}}_{i})={\sum }_{j=1}^{N-1}|{{\bf{X}}}_{i}(j+1)-{{\bf{X}}}_{i}(j)|=$ ${\sum }_{G\in {\mathscr{G}}}\Vert {{\bf{A}}}_{G}{{\bf{X}}}_{i}\Vert$. Using the duality concept^47^, we can establish the dual form of *TV*(**X**_*i*_)

5 $$TV({{\bf{X}}}_{i})=\sum _{G\in {\mathscr{G}}}\Vert {{\bf{A}}}_{G}{{\bf{X}}}_{i}\Vert =\sum _{G\in {\mathscr{G}}}\mathop{{\rm{\max }}}\limits_{{\Vert {z}_{G}\Vert }_{2}\le 1}\langle {z}_{G},{{\bf{A}}}_{G}{{\bf{X}}}_{i}\rangle =\mathop{{\rm{\max }}}\limits_{z\in {\mathscr{K}}}\langle z,{\bf{A}}{{\bf{X}}}_{i}\rangle ,$$

where ${z}_{G}\in {{\mathbb{R}}}^{|G|}$ is a vector of auxiliary variables associated with **A**_*G*_ and $z\in {\mathscr{K}}=\{z={[{z}_{{G}_{1}}^{T},\mathrm{...},{z}_{{G}_{|{\mathscr{G}}|}}^{T}]}^{T}:{\Vert {z}_{G}\Vert }_{2}\le 1,\forall G\in {\mathscr{G}}\}$. The **A** is the vertical concatenation of all the **A**_*G*_ matrices, that is

6 $${\bf{A}}=[\begin{array}{cccccc}-1 & 1 &  &  &  & \\  & -1 & 1 &  &  & \\  &  & \cdots  & \cdots  &  & \\  &  &  & -1 & 1 & \\  &  &  &  & -1 & 1\end{array}]$$

**Algorithm 1:**
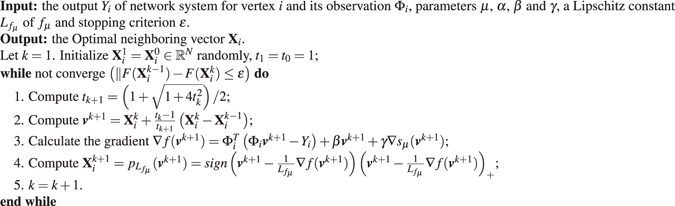
The main procedures of solving the problem (4)

The set ${\mathscr{K}}$ is the Cartesian product of unit balls in Euclidean space, therefore, a compact convex set. Before smoothing the TV penalty, we introduce the following important Lemma.

**Lemma 1** *Let* ${s}_{\mu }({{\bf{X}}}_{i})$ *be Nesterov’s smooth transform of* $TV({{\bf{X}}}_{i})$. *If* $M={{\rm{\max }}}_{z\in {\mathscr{K}}}{\Vert z\Vert }_{2}^{2}\mathrm{/2}$, then for all ${{\bf{X}}}_{i}\in {{\mathbb{R}}}^{p}$,

$${s}_{\mu }({{\bf{X}}}_{i})\le TV({{\bf{X}}}_{i})\le {s}_{\mu }({{\bf{X}}}_{i})+\mu M\mathrm{.}$$

By using Nesterov’s smothing technique, the smoothed TV penalty is formulated as

7 $${s}_{\mu }({{\bf{X}}}_{i})=\mathop{{\rm{\max }}}\limits_{z\in {\mathscr{K}}}\{\langle z,{\bf{A}}{{\bf{X}}}_{i}\rangle -\frac{\mu }{2}{\Vert z\Vert }_{2}^{2}\}=\langle {z}_{\mu }^{\ast }({{\bf{X}}}_{i}),{\bf{A}}{{\bf{X}}}_{i}\rangle -\frac{\mu }{2}{\Vert {z}_{\mu }^{\ast }({{\bf{X}}}_{i})\Vert }_{2}^{2},$$

where *μ* is a positive smothing parameter and

8 $${z}_{\mu }^{\ast }({{\bf{X}}}_{i})={\rm{\arg }}\mathop{\max }\limits_{z\in {\mathscr{K}}}\{\langle z,{\bf{A}}{{\bf{X}}}_{i}\rangle -\frac{\mu }{2}{\Vert z\Vert }_{2}^{2}\}\mathrm{.}$$

**Theorem 1** (Nesterov’s Theorem^27^). *Let the function s*_*μ*_ *be Nesterov’s smooth transform of a convex function TV penalty*. *We can obtain the following results*:

(1) *s*_*μ*_ is convex and differentiable with gradient $\nabla {s}_{\mu }({{\bf{X}}}_{i})={{\bf{A}}}^{T}{z}_{\mu }^{\ast }({{\bf{X}}}_{i})$ and $\nabla {s}_{\mu }({{\bf{X}}}_{i})$ is Lipschitz continuous with constant ${L}_{{s}_{\mu }}={\Vert {\bf{A}}\Vert }_{2}^{2}/\mu$, where ${\Vert {\bf{A}}\Vert }_{2}^{2}={\lambda }_{{\rm{\max }}}({{\bf{A}}}^{T}{\bf{A}})$ is the largest eigenvalue of **A** ^*T*^ **A**.

(2) For all ${{\bf{X}}}_{i}\in {{\mathbb{R}}}^{p}$, we have ${\mathrm{lim}}_{\mu \to 0}{s}_{\mu }({{\bf{X}}}_{i})=TV({{\bf{X}}}_{i})$.

The proof of Theorem 1 is obtained according to Lemma 1. According to the projection theorem^44^, we know that ${z}_{\mu }^{\ast }({{\bf{X}}}_{i})$ is the projection of $\frac{1}{\mu }{\bf{A}}{{\bf{X}}}_{i}$ onto the compact and convex space ${\mathscr{K}}$, that is

9 $${z}_{\mu }^{\ast }({{\bf{X}}}_{i})={P}_{{\mathscr{K}}}(\frac{1}{\mu }{\bf{A}}{{\bf{X}}}_{i})=\prod _{G\in {\mathscr{G}}}{P}_{{{\mathscr{K}}}_{G}}(\frac{1}{\mu }{{\bf{A}}}_{G}{{\bf{X}}}_{i}),$$

where Π is the Cartesian product of a set and ${P}_{{{\mathscr{K}}}_{G}}(\cdot )$ is the projection onto each compact set ${{\mathscr{K}}}_{G}$ defined as

$${P}_{{{\mathscr{K}}}_{G}}(x)=\frac{x}{{\rm{\max }}({\Vert x\Vert }_{2},1)}=\{\begin{array}{cc}x, & if\,{\Vert x\Vert }_{2}\le 1\\ \frac{x}{{\Vert x\Vert }_{2}}, & otherwise\mathrm{.}\end{array}$$

### Optimization

After smoothing by Nesterov’s technique, the proximal operator of *s*_*μ*_ becomes easily computed. We obtain a new optimization problem by using *s*_*μ*_(**X**_*i*_) to replace the TV penalty of the problem (4):

10 $$\mathop{{\rm{\min }}}\limits_{{{\bf{X}}}_{i}}{F}_{\mu }({{\bf{X}}}_{i})\mathop{=}\limits^{\Delta }{f}_{\mu }({{\bf{X}}}_{i})+\lambda {\Vert {{\bf{X}}}_{i}\Vert }_{1}=\frac{1}{2}{\Vert {y}_{i}-{\Phi }_{i}{{\bf{X}}}_{i}\Vert }_{2}^{2}+\frac{\beta }{2}{\Vert {{\bf{X}}}_{i}\Vert }_{2}^{2}+\lambda {\Vert {{\bf{X}}}_{i}\Vert }_{1}+\gamma {s}_{\mu }({{\bf{X}}}_{i}),$$

where ${f}_{\mu }({{\bf{X}}}_{i})={\Vert {y}_{i}-{\Phi }_{i}{{\bf{X}}}_{i}\Vert }_{2}^{2}+\frac{\beta }{2}{\Vert {{\bf{X}}}_{i}\Vert }_{2}^{2}+\gamma {s}_{\mu }({{\bf{X}}}_{i})$ is a smooth convex function, i.e., continuously differentiable with Lipschitz continuous gradient constant ${L}_{{f}_{\mu }}$

$$\Vert \nabla {f}_{\mu }(u)-\nabla {f}_{\mu }(v)\Vert \le {L}_{{f}_{\mu }}\Vert u-v\Vert ,\forall u,v\in {{\mathbb{R}}}^{d},$$

and *s*_*μ*_(**X**_*i*_) is Nesterov’s smoothing function of *TV*(**X**_*i*_) and defined as Equation (7). Motivated by the idea of FISTA^42^, we adopt the following quadratic model as the approximation of *F*_*μ*_(**X**_*i*_) at a given point *v*

11 $${Q}_{{L}_{{f}_{\mu }}}({{\bf{X}}}_{i},v)={f}_{\mu }(v)+\langle {{\bf{X}}}_{i}-v,\nabla {f}_{\mu }(v)\rangle +\frac{{L}_{{f}_{\mu }}}{2}{\Vert {{\bf{X}}}_{i}-v\Vert }^{2}+\lambda {\Vert {{\bf{X}}}_{i}\Vert }_{1},$$

which admits a unique minimizer

12 $${p}_{{L}_{{f}_{\mu }}}(v)={\rm{\arg }}\mathop{{\rm{\min }}}\limits_{{{\bf{X}}}_{i}}\,{Q}_{L}({{\bf{X}}}_{i},v)=\text{arg}\mathop{\min }\limits_{{{\bf{X}}}_{i}}(\lambda \parallel {{\bf{X}}}_{i}{\parallel }_{1}+\frac{1{L}_{{f}_{\mu }}}{2}{\Vert {{\bf{X}}}_{i}-(v-\frac{1}{{L}_{{f}_{\mu }}}\nabla f(v))\Vert }^{2}),$$

where $\nabla f(v)={\Phi }_{i}^{T}({\Phi }_{i}v-{Y}_{i})+\beta v+\gamma \nabla {s}_{\mu }(v)$. Note that ${p}_{{L}_{{f}_{\mu }}}(v)$ is the well known component-wise soft thresholding operator

$${({p}_{{L}_{{f}_{\mu }}}(v))}_{i}=sign({{\boldsymbol{u}}}_{i})\,{(|{{\boldsymbol{u}}}_{i}|-\lambda )}_{+},$$

where $u=v-\frac{1}{{L}_{{f}_{\mu }}}\nabla f(v)$ and (·)_+_ = max(·, 0).

The main procedure of our method is described in Algorithm 1. Denote *t*_0_=0, as FISTA, we generate two vectors $({v}^{k+1},{{\bf{X}}}_{i}^{k+1})$ to minimize the smoothed model in (10) at *k+*1 iteration:

13 $$\begin{array}{l}{v}^{k+1}={{\bf{X}}}_{i}^{k}+\frac{{t}_{k}-1}{{t}_{k+1}}({{\bf{X}}}_{i}^{k}-{{\bf{X}}}_{i}^{k-1})\,and\,{{\bf{X}}}_{i}^{k+1}={p}_{{L}_{{f}_{\mu }}}({v}^{k+1}),\end{array}$$

where ${t}_{k+1}=(1+\sqrt{1+4{t}_{k}^{2}})\mathrm{/2}$. By using this iterative method to minimize the smoothed model in (10), it will converge to ${{\bf{X}}}_{i}^{\ast }$, which is the minimum of *F*_*μ*_(**X**_*i*_). Moreover, ${\Vert {{\bf{X}}}_{i}\Vert }_{1}$ is a strong convex function, the convergence rate is guaranteed by

14 $${F}_{\mu }({{\bf{X}}}_{i}^{k})-F({{\bf{X}}}_{i}^{\ast })\le \frac{2{L}_{{f}_{\mu }}{\Vert {{\bf{X}}}_{i}^{0}-{{\bf{X}}}_{i}^{\ast }\Vert }^{2}}{{(k+1)}^{2}},$$

where *k* is the number of iterations. Meanwhile, by the duality gap theory and theoretical results in refs 27, 42, 48, we can obtain the following important result:

15 $$\mathop{\mathrm{lim}}\limits_{k\to \infty }{F}_{\mu }({{\bf{X}}}_{i}^{k})=F({{\bf{X}}}_{i}^{\ast }),$$

and the number of iterations is upper bounded by

16 $$(\frac{\sqrt{8{\Vert {\bf{A}}\Vert }_{2}^{2}M{\gamma }^{2}}}{\varepsilon }+\frac{\sqrt{2{L}_{{f}_{\mu }}}}{\sqrt{\varepsilon }}){\Vert {{\bf{X}}}_{i}^{0}-{{\bf{X}}}_{i}^{\ast }\Vert }_{2},$$

provided that ${F}_{\mu }({{\bf{X}}}_{i}^{k})-F({{\bf{X}}}_{i}^{\ast }) < \varepsilon$. The more details of proof are referred to refs 27, 42 and 48.

### ﻿Data ﻿Av﻿ailability

All original data are available from the website FigShare https://figshare.com/articles/adjnoun_gml/4883708.﻿
