# Author Correction: A Robust Method for Inferring Network Structures

**DOI:** 10.1038/s41598-018-21956-z

**Published:** 2018-03-08

**Authors:** Yang Yang, Tingjin Luo, Zhoujun Li, Xiaoming Zhang, Philip S. Yu

**Affiliations:** 1 School of Computer Science and Engineering, Beihang University, Beijing, 100191 China; 2 Department of Computer Science, University of Illinois at Chicago, Chicago, 60607 United States; 3 College of Science, National University of Defense Technology, Changsha, Hunan 410073 China; 4 Department of Computational Medicine and Bioinformatics, University of Michigan, Ann Arbor, MI 48109 USA

Correction to: *Scientific Reports* 10.1038/s41598-017-04725-2, published online 12 July 2017

This Article contains typographical errors in the Acknowledgements section.

“This work was supported by the National Natural Science Foundation of China (Grand Nos 61370126, 61672081, U1636211),”

should read:

“This work was supported by the National Natural Science Foundation of China (Grand Nos U1636211, 61370126, 61672081),”

